# Supplementary material for: Heavy-boundary mode patterning and dynamics of topological phonons in polymer chains and supramolecular lattices on surfaces
Source: Nat Commun. 2024 Dec 11;15:10674. doi: 10.1038/s41467-024-54511-8 (PMC11634973; doi:10.1038/s41467-024-54511-8)
Supplement: Supplementary file 1 — Supplementary Information [file 41467_2024_54511_MOESM1_ESM.pdf]

Supplementary Information:

Heavy-boundary mode patterning and dynamics of topological phonons in polymers and supramolecular lattices on surfaces

*José D. Cojal González,[1] Jakub Rondomanski,[2] Konrad Polthier,[2] Jürgen P. Rabe[1] and Carlos-Andres Palma[1,3]**

[1] *Department of Physics & IRIS Adlershof, Humboldt-Universität zu Berlin, Berlin, Germany*

[2] *Department of Mathematics and Computer Science, Freie Universität Berlin, Berlin, Germany*

[3] *Institute of Physics, Chinese Academy of Sciences, Beijing, P. R. China*

Supplementary Methods

Dynamical Matrix Approach

We used the dynamical matrix approach[1] to describe the linear response of our simplified mechanical systems. A *d*-dimensional frame consisting of N point masses connected by N_C springs is fully described by the compatibility matrix C, relating the Nd-dimensional vector of sites displacements $\boldsymbol{u}$ to the N_C-dimensional vector of bond elongations $\boldsymbol{e}$:

$$\mathrm{C} \cdot \boldsymbol{u} = \boldsymbol{e} \qquad (1)$$

The normal modes of the system are the eigenvalues of the dynamical matrix $\mathbf{D}$, which is given by:

$$\mathrm{D} = \mathrm{M}^{-1}\mathrm{C}^* \, \kappa \, \mathrm{C}\,, \qquad (2)$$

where κ is the $N_C \times N_C$ diagonal matrix of spring constants. C^* denotes the conjugate transpose matrix of C. M^{-1} is the inverse of the diagonal matrix of masses. Bond elongations are obtained using, $\boldsymbol{e_n} = \hat{e}_n \cdot (\boldsymbol{u}_j - \boldsymbol{u}_i)$, where $\hat{e}_n$ is the unit vector pointing from site i to j in the undistorted lattice.

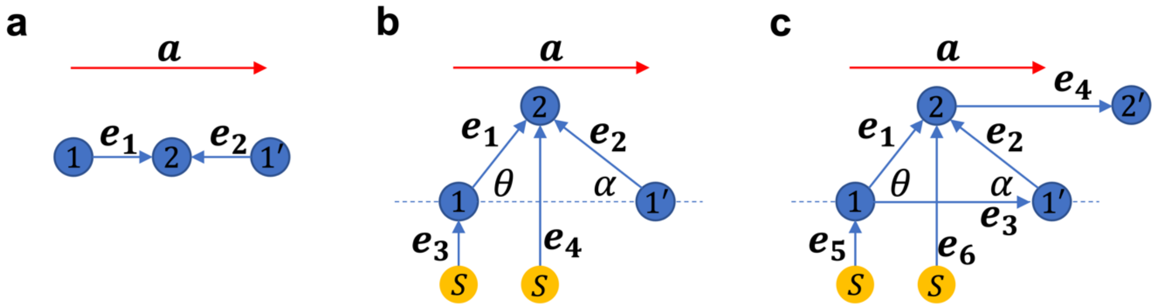

Supplementary Figure 1: **a.** pSSH model showing the elongation vectors $\boldsymbol{e_n}$ for the unit cell characterized by the unit cell vector $\boldsymbol{a}$ vector. **b.** aSSH model showing the elongation vectors $\boldsymbol{e_n}$ for the unit cell characterized by the unit cell vector $\boldsymbol{a}$ vector. **c.** daSSH model showing the elongation vectors $\boldsymbol{e_n}$ for the unit cell characterized by the unit cell vector $\boldsymbol{a}$ vector. The substrates mases *S* are fixed. We assume all masses are equal to m.

For the aSSH case:

$$\boldsymbol{e_1} = \hat{e}_1 \cdot (\boldsymbol{u}_1 - \boldsymbol{u}_2)$$

$$\boldsymbol{e_2} = \hat{e}_2 \cdot \big(\boldsymbol{u}_2 - \boldsymbol{u}_1(\boldsymbol{r} + \boldsymbol{a})\big)$$

$$\boldsymbol{e_3} = \hat{e}_3 \cdot \boldsymbol{u}_1$$

$$\boldsymbol{e_4} = \hat{e}_4 \cdot \boldsymbol{u}_2$$

In this way we can construct the connectivity matrix:

$$\begin{pmatrix} e_1 \\ e_2 \\ e_3 \\ e_4 \end{pmatrix} = \begin{pmatrix} \hat{e}_{1x} & -\hat{e}_{1x} & \hat{e}_{1y} & -\hat{e}_{1y} \\ -e^{i\boldsymbol{k}\cdot\boldsymbol{a}}\hat{e}_{2x} & \hat{e}_{2x} & e^{i\boldsymbol{k}\cdot\boldsymbol{a}}\hat{e}_{2y} & -\hat{e}_{2y} \\ 0 & 0 & 1 & 0 \\ 0 & 0 & 0 & 1 \end{pmatrix} \begin{pmatrix} u_{1x} \\ u_{2x} \\ u_{1y} \\ u_{2y} \end{pmatrix} \quad (3)$$

where $\boldsymbol{a}$ is the unit vector. In terms of the angles of Scheme 1 our connectivity matrix reads:

$$\mathrm{C} = \begin{pmatrix} \cos\theta & -\cos\theta & \sin\theta & -\sin\theta \\ -e^{i\boldsymbol{k}\cdot\boldsymbol{a}}\cos\alpha & \cos\alpha & e^{i\boldsymbol{k}\cdot\boldsymbol{a}}\sin\alpha & -\sin\alpha \\ 0 & 0 & 1 & 0 \\ 0 & 0 & 0 & 1 \end{pmatrix} \quad (4)$$

We consider the case where all masses are equal to $m = 1$. Then equation 2 allow us to construct the dynamical matrix:

$$\mathrm{D_{aSSH}} = \begin{pmatrix} \cos\theta & -e^{-i\boldsymbol{k}\cdot\boldsymbol{a}}\cos\alpha & 0 & 0 \\ -\cos\theta & \cos\alpha & 0 & 0 \\ \sin\theta & e^{-i\boldsymbol{k}\cdot\boldsymbol{a}}\sin\alpha & 1 & 0 \\ -\sin\theta & -\sin\alpha & 0 & 1 \end{pmatrix} \begin{pmatrix} \kappa_1 & 0 & 0 & 0 \\ 0 & \kappa_2 & 0 & 0 \\ 0 & 0 & \kappa_3 & 0 \\ 0 & 0 & 0 & \kappa_4 \end{pmatrix} \begin{pmatrix} \cos\theta & -\cos\theta & \sin\theta & -\sin\theta \\ -e^{i\boldsymbol{k}\cdot\boldsymbol{a}}\cos\alpha & \cos\alpha & e^{i\boldsymbol{k}\cdot\boldsymbol{a}}\sin\alpha & -\sin\alpha \\ 0 & 0 & 1 & 0 \\ 0 & 0 & 0 & 1 \end{pmatrix}$$

$$\mathrm{D}_{aSSH} = \begin{pmatrix} D_{11} & D_{12} & D_{13} & D_{14} \\ D_{21} & D_{22} & D_{23} & D_{24} \\ D_{31} & D_{32} & D_{33} & D_{34} \\ D_{41} & D_{42} & D_{43} & D_{44} \end{pmatrix} \quad (5)$$

And the components:

$$D_{11} = D_{22} = \kappa_1 \cos^2\theta + \kappa_2 \cos^2\alpha$$

$$D_{12} = -\kappa_1 \cos^2\theta - \kappa_2 e^{-i\boldsymbol{k}\cdot\boldsymbol{a}} \cos^2\alpha$$

$$D_{13} = D_{24} = D_{31} = D_{42} = \kappa_1 \sin\theta\cos\theta - \kappa_2 \sin\alpha\cos\alpha$$

$$D_{14} = D_{32} = -\kappa_1 \sin\theta\cos\theta + \kappa_2 e^{-i\boldsymbol{k}\cdot\boldsymbol{a}} \sin\alpha\cos\alpha$$

$$D_{21} = -\kappa_1 \cos^2\theta - \kappa_2 e^{i\boldsymbol{k}\cdot\boldsymbol{a}} \cos^2\alpha$$

$$D_{23} = D_{41} = -\kappa_1 \sin\theta\cos\theta + \kappa_2 e^{i\boldsymbol{k}\cdot\boldsymbol{a}} \sin\alpha\cos\alpha$$

$$D_{33} = \kappa_1 \cos^2\theta + \kappa_2 \cos^2\alpha + \kappa_3$$

$$D_{34} = -\kappa_1 \sin^2\theta - \kappa_2 e^{-i\boldsymbol{k}\cdot\boldsymbol{a}} \sin^2\alpha$$

$$D_{43} = -\kappa_1 \sin^2\theta - \kappa_2 e^{i\boldsymbol{k}\cdot\boldsymbol{a}} \sin^2\alpha$$

$$D_{44} = \kappa_1 \cos^2\theta + \kappa_2 \cos^2\alpha + \kappa_4$$

Equation 5 can be can also written as a Hermitian matrix:

$$\mathrm{D}_{aSSH} = \begin{pmatrix} D_{11} & D_{12} & D_{13} & D_{14} \\ D_{12}^* & D_{11} & D_{14}^* & D_{13} \\ D_{13} & D_{14} & D_{11} + \kappa_3 & D_{34} \\ D_{14}^* & D_{13} & D_{34}^* & D_{11} + \kappa_4 \end{pmatrix} \quad (6)$$

We can prove the time-reversal symmetry of this matrix by the operation $U_T D_{aSSH} + D_{aSSH} U_T$,[2] where

$$U_T = \begin{pmatrix} 1 & 0 & 0 & 0 \\ 0 & -1 & 0 & 0 \\ 0 & 0 & 1 & 0 \\ 0 & 0 & 0 & -1 \end{pmatrix} \mathrm{X}$$

is the time-reversal unitary operator and X is the complex conjugate operator.

$$U_T D_{aSSH} + D_{aSSH} U_T = 0$$

Further, for the daSSH model, the connectivity matrix is given by:

$$\mathrm{C} = \begin{pmatrix} \cos\theta & -\cos\theta & \sin\theta & -\sin\theta & 0 & 0 \\ -e^{i\boldsymbol{k}\cdot\boldsymbol{a}}\cos\alpha & \cos\alpha & e^{i\boldsymbol{k}\cdot\boldsymbol{a}}\sin\alpha & -\sin\alpha & 0 & 0 \\ 1-e^{i\boldsymbol{k}\cdot\boldsymbol{a}} & 0 & 0 & 0 & 0 & 0 \\ 0 & 1-e^{i\boldsymbol{k}\cdot\boldsymbol{a}} & 0 & 0 & 0 & 0 \\ 0 & 0 & 0 & 0 & 1 & 0 \\ 0 & 0 & 0 & 0 & 0 & 1 \end{pmatrix} \quad (7)$$

And the dynamical Hermitian matrix:

$$\mathrm{D}_{daSSH} = \begin{pmatrix} D_{11} & D_{12} & D_{13} & D_{14} & 0 & 0 \\ D_{12}^* & D_{11} & D_{14}^* & D_{13} & 0 & 0 \\ D_{13} & D_{14} & D_{33} & D_{34} & 0 & 0 \\ D_{14}^* & D_{13} & D_{34}^* & D_{33} & 0 & 0 \\ 0 & 0 & 0 & 0 & \kappa_5 & 0 \\ 0 & 0 & 0 & 0 & 0 & \kappa_6 \end{pmatrix} \quad (8)$$

where the components are:

$$D_{11} = D_{22} = \kappa_1 \cos^2\theta + \kappa_2 \cos^2\alpha + 2\kappa_3(1-\cos\boldsymbol{k}\cdot\boldsymbol{a})$$

$$D_{12} = -\kappa_1\cos^2\theta - \kappa_2 e^{-i\boldsymbol{k}\cdot\boldsymbol{a}}\cos^2\alpha$$

$$D_{13} = \kappa_1 \sin\theta\cos\theta - \kappa_2\sin\alpha\cos\alpha$$

$$D_{14} = -\kappa_1\sin\theta\cos\theta + \kappa_2 e^{-i\boldsymbol{k}\cdot\boldsymbol{a}}\sin\alpha\cos\alpha$$

$$D_{33} = \kappa_1\sin^2\theta + \kappa_2\sin^2\alpha$$

$$D_{34} = -\kappa_1\sin^2\theta - \kappa_2 e^{-i\boldsymbol{k}\cdot\boldsymbol{a}}\sin^2\alpha$$

Similarly, we can prove the time-reversal symmetry of this matrix by performing the matrix operation $U_T D_{daSSH} + D_{daSSH} U_T$,[2] where

$$U_S = \begin{pmatrix} 1 & 0 & 0 & 0 & 0 & 0 \\ 0 & -1 & 0 & 0 & 0 & 0 \\ 0 & 0 & 1 & 0 & 0 & 0 \\ 0 & 0 & 0 & -1 & 0 & 0 \\ 0 & 0 & 0 & 0 & 1 & 0 \\ 0 & 0 & 0 & 0 & 0 & -1 \end{pmatrix} \mathrm{X}$$

is the time-reversal unitary operator.

$$U_T D_{aSSH} + D_{aSSH} U_T = 0$$

Density Functional Tight Binding (DFTB) Power Spectrum

The power spectrum of the system was obtained through the fast Fourier Transform (FFT) of the velocity autocorrelation function, derived from molecular dynamics (MD) simulations conducted on the Born-Oppenheimer ground state energy surface.

Molecular dynamics simulations were run for 100 ps with a time step of 0.5 fs. Velocity Verlet[3] algorithm was employed as the integrator to ensure stable and accurate propagation of the atomic trajectories. To control the temperature, the Nose-Hoover[4,5] thermostat was applied, maintaining the system at a constant temperature of 10 K with a coupling strength parameter of 1.11 fs.

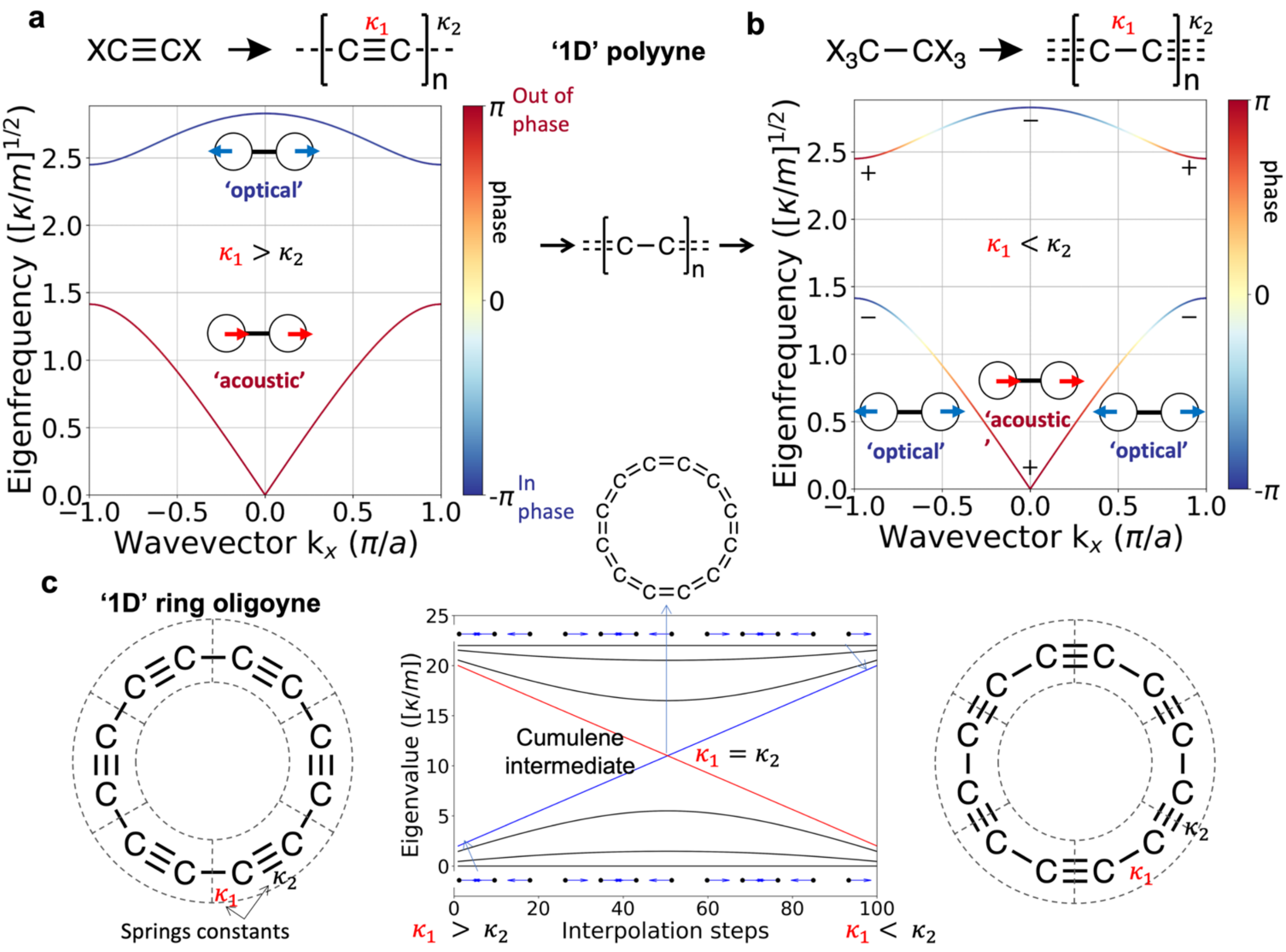

Supplementary Figure 2. a. Trivial case of the Su-Schrieffer-Heeger phonon analogue (pSSH) model (trivial polymer) consists of monomers of two triple bonded carbon atoms that are single bonded to their neighbours. In the trivial band structure, bands can be classified as optical and acoustic in terms of the inner product between vibrational displacement in one unit-cell. When the inner product is positive, the atoms move in-phase while otherwise they move out-of-phase. For the trivial polymer, the lower energy vibrations move in-phase (acoustic branch), while the higher energy vibrations move out-of-phase (optical branch). **b.** The topological case of the SSH model (topological polymer) occurs by exchanging the strong and weak springs, which produces an inversion in the phase of the branches along the unit cell wavevector, without affecting the vibrational spectrum. The outermost in-phase vibration is shifted to a higher energy and, at the same time the outer-most out-of-phase vibration is shifted to lower energies. The net effect is that the phase of both branches is inverted when spanning the symmetrical wavelength interval $\left[-\frac{\pi}{a}, \frac{\pi}{a}\right]$ or first Brillouin zone, with associated winding number (Supplementary Figure 3). **c**. Eigenvalue spectra obtained upon switching values of κ_1 and κ_2 from 10 to 1 in a periodic system of 12 masses. The inset images show the (real space) displacement vectors for the eigenmode transported from low to high frequency (blue line). The spring chain can be represented as mechanical oligoyne, wherein the spring change entails moving through the cumulene phase. This interpolation leads to an

eigenvalue inversion which becomes meaningful at the band whereby two pSSH phases emerge separated by the cumulene phase.

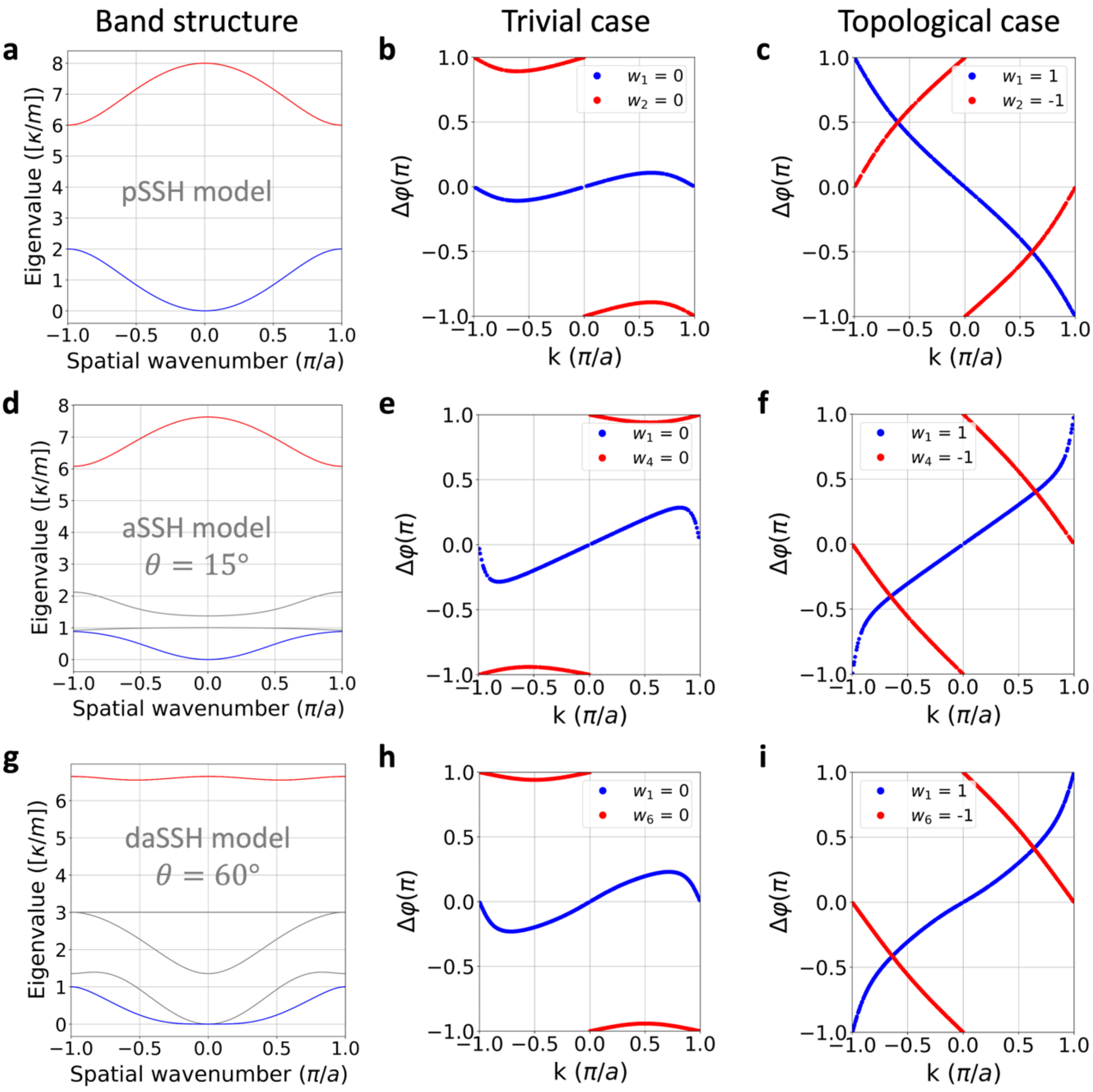

Supplementary Figure 3. Band structures (**a,d,g**) and geometric phases for the trivial **(b,e,h**) and topological (**c,f,i**) phases of pSSH (**a,b,c**), aSSH (**d,e,f**) and daSSH (**g,h,i**) models. Winding numbers w around the torus in the Brillouin zone are given for the selected bands.

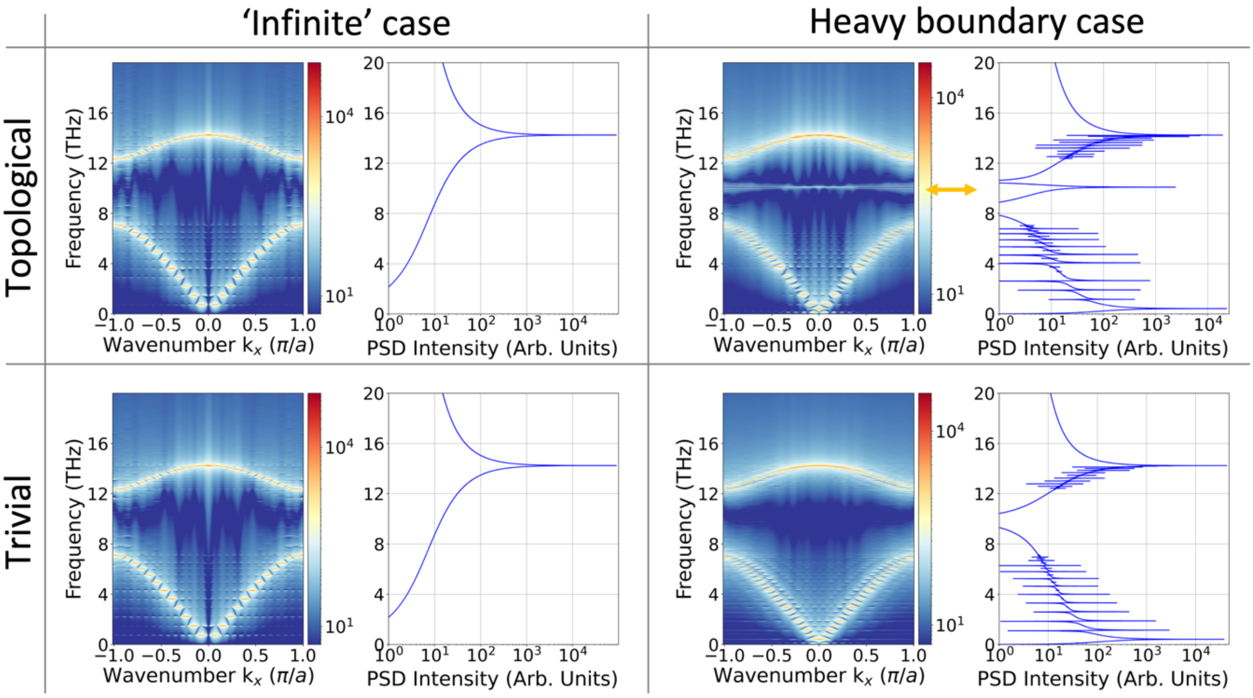

Supplementary Figure 4. 1D phonon Su-Schrieffer-Heeger (pSSH) model, consisting on 52 mass chain of alternating spring constants κ_1 and κ_2. For the topological (trivial) case κ_1 = 3000kJ/mol·nm^2 (1000kJ/mol·nm^2) and κ_2 = 1000kJ/mol·nm^2 (3000kJ/mol·nm^2). For the heavy boundary case the first and last mass are equal to 100au and the rest are 1au. For the infinite case all the masses are equal to 1au.

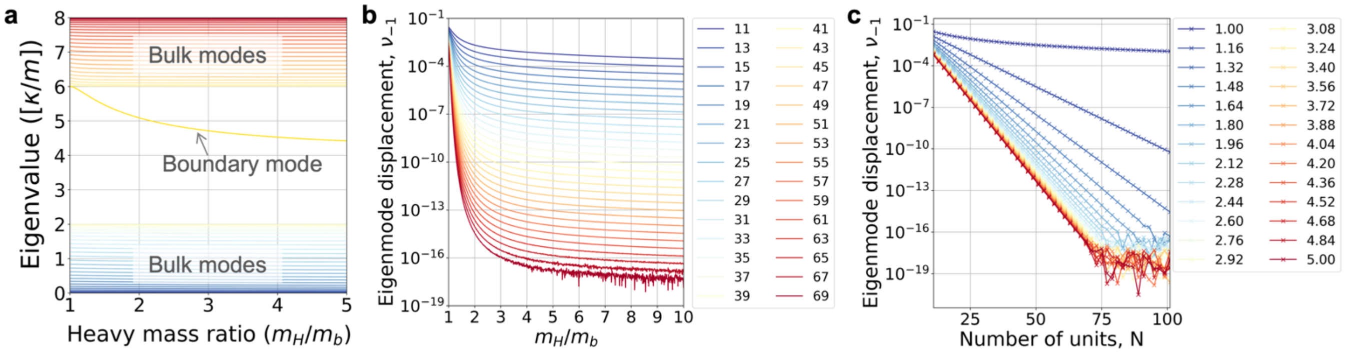

Supplementary Figure 5. a. Eigenvalues for a Su-Schrieffer-Heeger phonon analogue (pSSH) chain of 101 units as the mass of the first unit (heavy mass or m_H) is increased, the rest of the mases, m_b, are equal to 1.0. A topological boundary mode (TBM) emerges between the bulk modes when $m_H > m_b$. **b.** The magnitude of the eigenmode displacement in the unit farthest away from the heavy one, ν_{-1}, shows a sharp decay as the ratio m_H/m_b increases. **c.** The eigendisplacement ν_{-1} decays exponentially with increasing number of units N. The relations are described in equation 1.

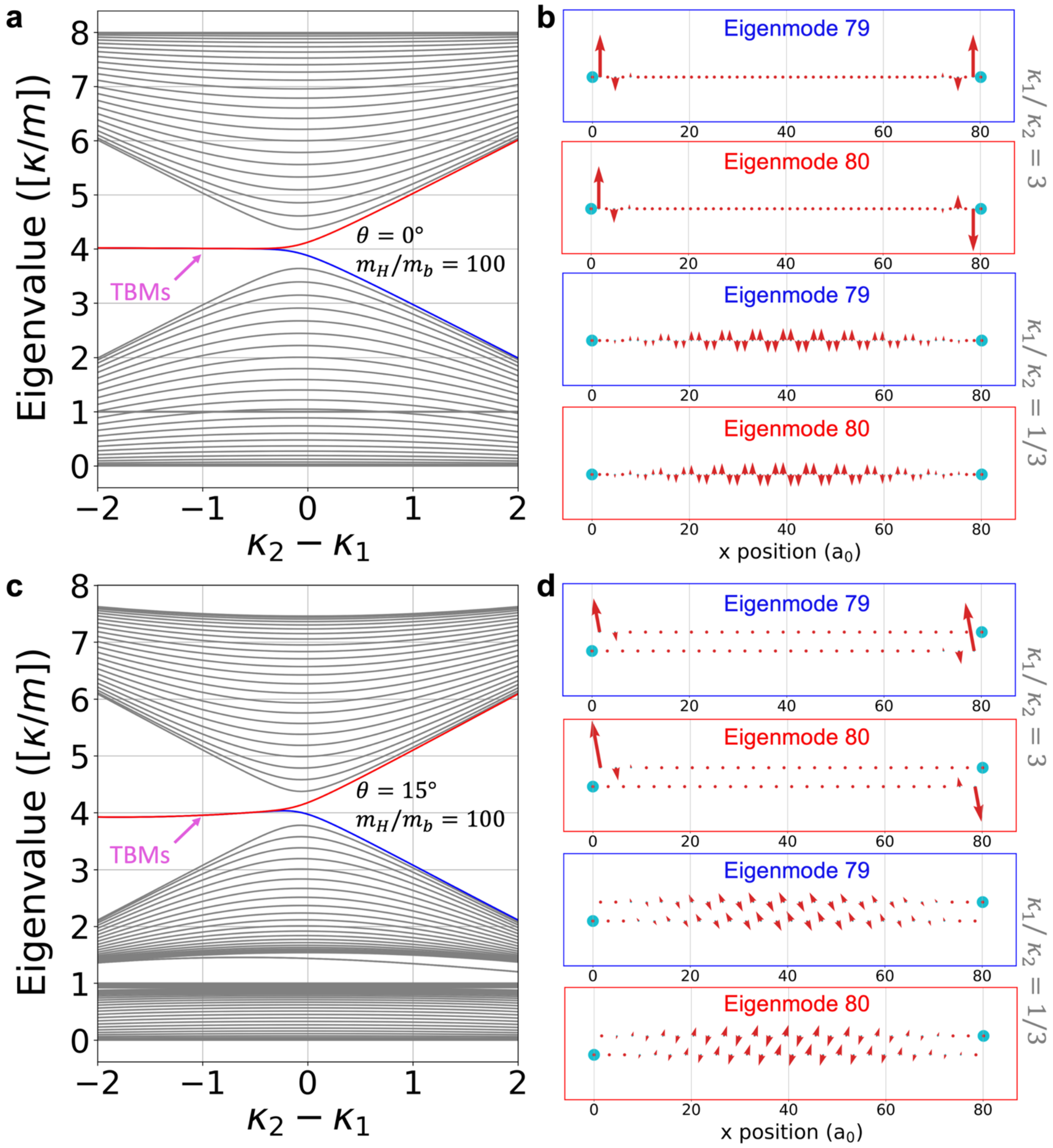

Supplementary Figure 6. Eigenvalue spectra when weak and strong springs are exchanged for a system of 52 pearls described by the adsorbed Su-Schrieffer-Heeger (aSSH) model for $\theta = 0°$ (**a**) and $\theta = 15°$ (**c**). The starting (ending) value of κ_1, $\kappa_2 = 3, 1\ (1, 3)$ and $\kappa_3 = 1$. In both spectra, a double degenerate (eigenmodes 79 and 80) topological boundary mode (TBM) is recognized when $\kappa_1 > \kappa_2$. This TBM splits in two bulk modes (one optical and one acoustical) when $\kappa_1 < \kappa_2$. **b,d.** Eigenmode maps for the eigenmodes 79 and 80 showing exponential localization next to the heavy mass when $\kappa_1/\kappa_2 = 3$ and delocalization into the bulk when $\kappa_1/\kappa_2 = 1/3$. The longitudinal eigenmode displacement in **b** are shown transversal to the actual movement for convenience.

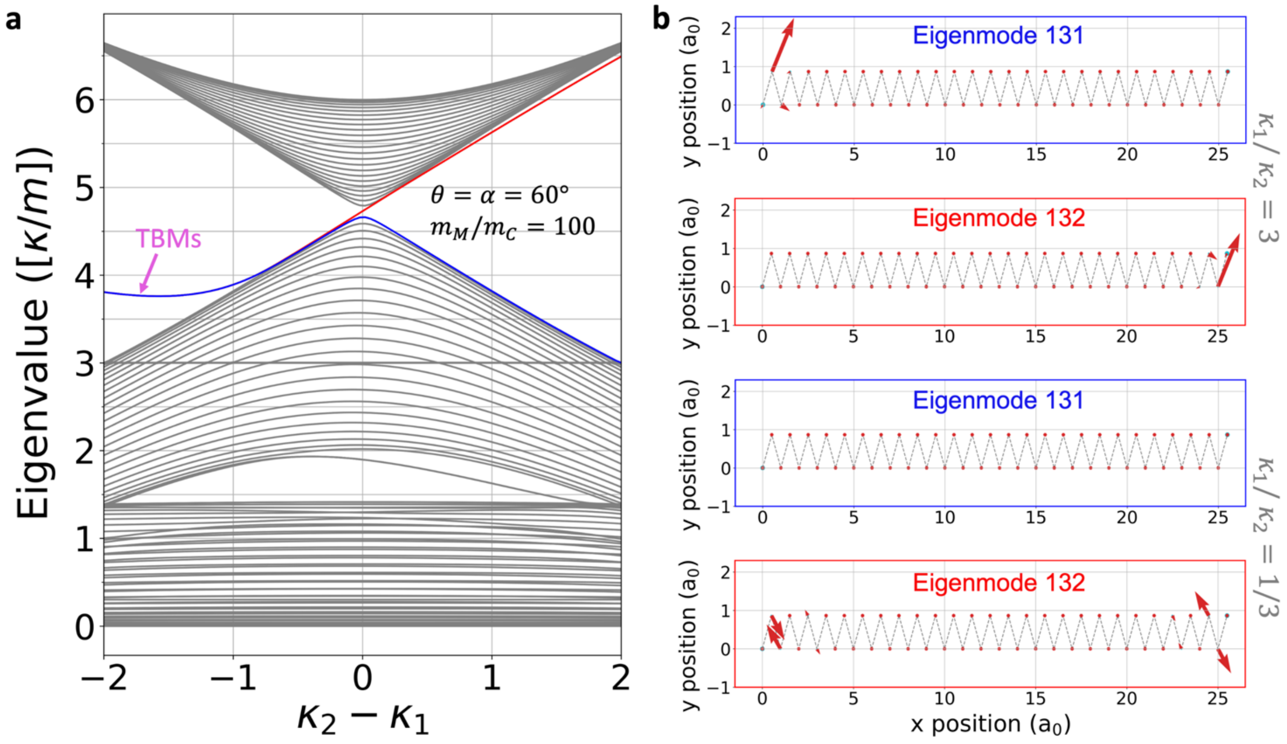

Supplementary Figure 7. a. Eigenvalue spectra when weak and strong springs are exchanged for a system of 52 pearls described by the double-chain adsorbed Su-Schrieffer-Heeger (daSSH) model. The starting (ending) value of $\kappa_1, \kappa_2 = 3, 1\ (1, 3)$, $\kappa_3 = 3$ and $\kappa_4 = \kappa_5 = 0.5$. When $\kappa_1 > \kappa_2$ a double degenerate (eigenmodes 131 and 132) topological boundary mode (TBM) is recognized. For $\kappa_1 < \kappa_2$, the TBM splits in two bulk modes, one acoustical (131 in blue) and one optical (132 in red). **b.** Eigenmode maps for the eigenmodes 131 and 132 showing exponential localization next to the heavy mass when $\kappa_1/\kappa_2 = 3$. When $\kappa_1/\kappa_2 = 1/3$, the acoustical eigenmode 131 is delocalized into the bulk, while the optical one into a high energy out-of-phase eigenmode.

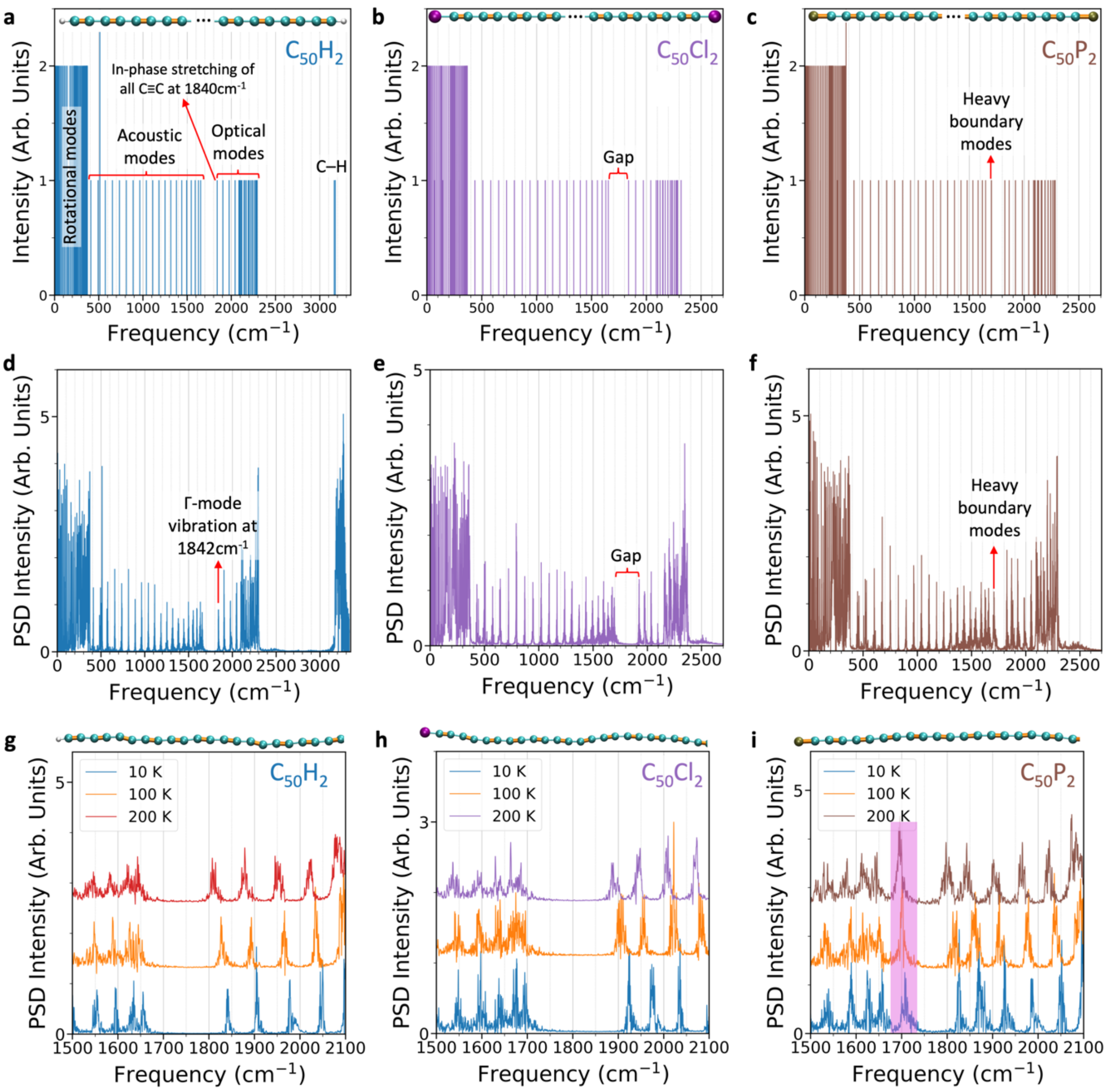

Supplementary Figure 8. Normal mode analysis (NMA, **a-c**) and power spectra (PSD, **d-f**) for 1D polyyne chains, consisting on 50 carbon atoms with H (**a,d,g**) , Cl (**b,e,h**) and P (**c,f,i**) as termini atoms. Simulations at a quantum level were performed using DFTB. The in-phase stretching of all triple bonds of $C_{50}H_2$ at 1840cm^{-1} (**a**) is reproduced in the PSD at 10 K (**d**), within the expected range for Raman Γ-mode vibration for a chain of this length[6]. Both NMA and PSD for $C_{50}H_2$ and $C_{50}Cl_2$ show a gap between acoustic and optical branches. When a heavier atom such as phosphorus is attached via triple bond to the carbon chain, $C_{50}P_2$, a heavy boundary mode appears within the gap. The inset in **c** shows that the eigendisplacement (depicted transversally for convenience) of the boundary modes (numbers 131 and 132) is exponentially localized in the mass next to the heavy one. The boundary mode in $C_{50}P_2$ can be identified at temperatures between 10 and 200 K (highlighted region in **g**). Snapshots after 100 ps of DFTB MD simulations at 200 K are depicted as insets in **g** ($C_{50}H_2$), **h** ($C_{50}Cl_2$) and **i** ($C_{50}P_2$).

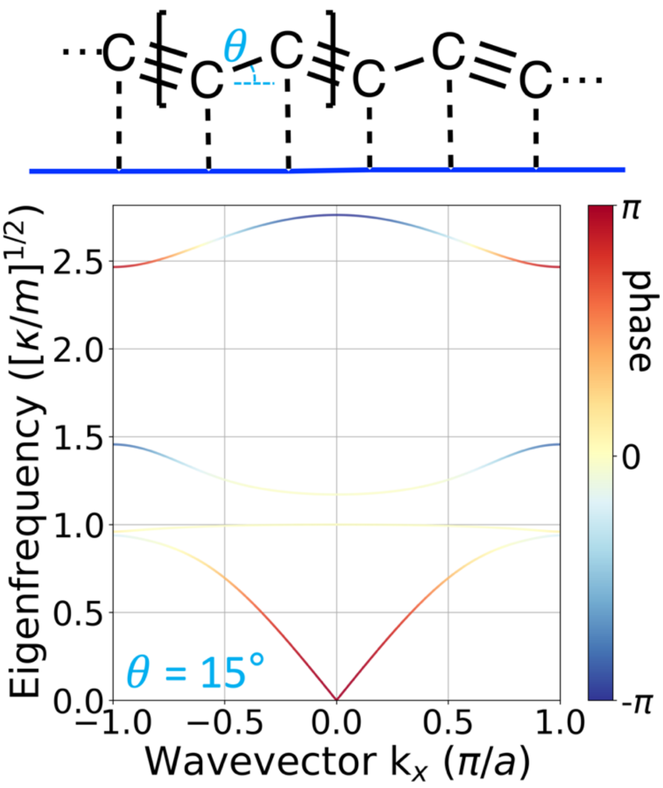

Supplementary Figure 9. aSSH model at equilibrium for a finite angle θ=15° between the masses and the substrate.

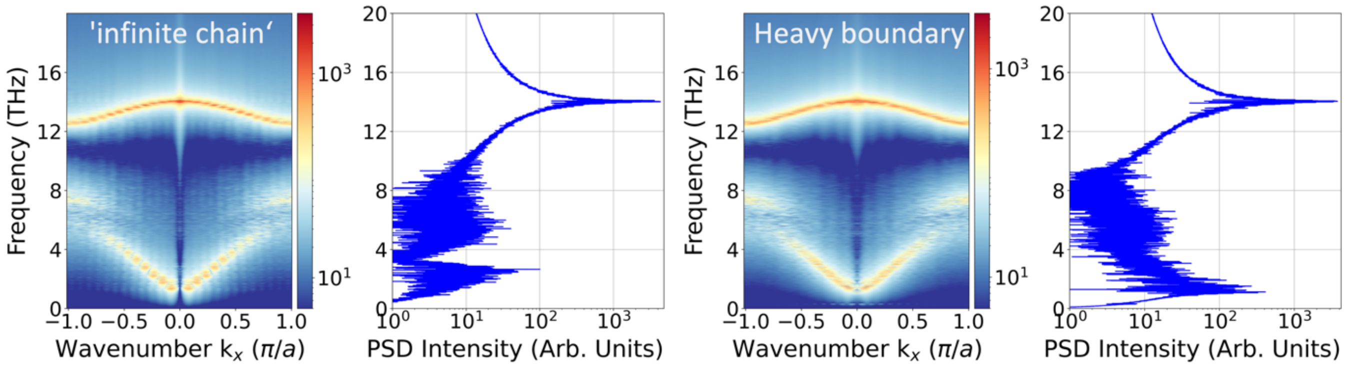

Supplementary Figure 10. Trivial case of aSSH model (Figure 3 of main text). The system consists of 52 mass chain of alternating spring constants κ_1 and κ_2. For the topological (trivial) case κ_1 = 3000kJ/mol·nm (1000kJ/mol·nm) and κ_2 = 1000kJ/mol·nm (3000kJ/mol·nm), while κ_3 = 8000kJ/mol·nm for all masses. For the heavy boundary case, the first and last masses are equal to 100au and the rest are 1au. For the infinite case, all the masses are equal to 1au. The substrate is harmonically restricted in 3D by a κ_{3D} = 10^6 kJ/mol·nm in all three dimensions. The masses are restricted to move in a plane with a harmonic restriction of κ_y = 10^6 kJ/mol·nm.

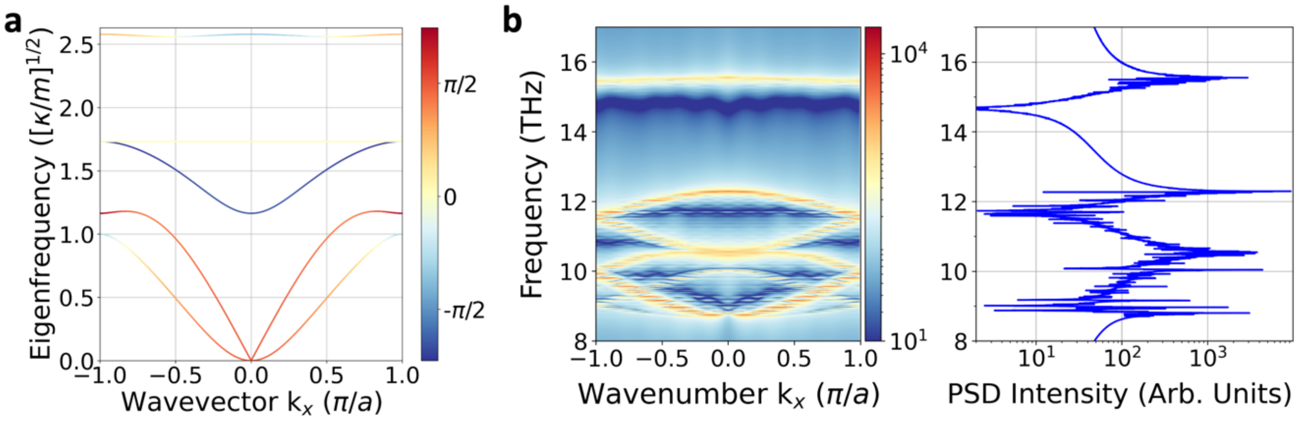

Supplementary Figure 11. Trivial case of daSSH model (Figure 4 of main text). **a.** The dynamical band structure on the left is generated using the parameters $\kappa_1 = 1,\ \kappa_2 = 3,\ \kappa_3 = 3$ and $\kappa_4 = \kappa_5 = 0.5,\ \theta = \alpha = 60°$ and $m_c = 1$. **b.** Molecular dynamics (MD) phonon band structure of the heavy boundary case for a system of 52 masses. Bond parameters: κ_1 = 1000kJ/mol·nm (3000kJ/mol·nm in Figure 3) and κ_2 = 3000kJ/mol·nm (1000kJ/mol·nm in Figure 3), κ_3 = 7000kJ/mol·nm, and $\kappa_4 = \kappa_5$ = 500kJ/mol·nm. The first and last masses are equal to 100au and the rest are 1au.

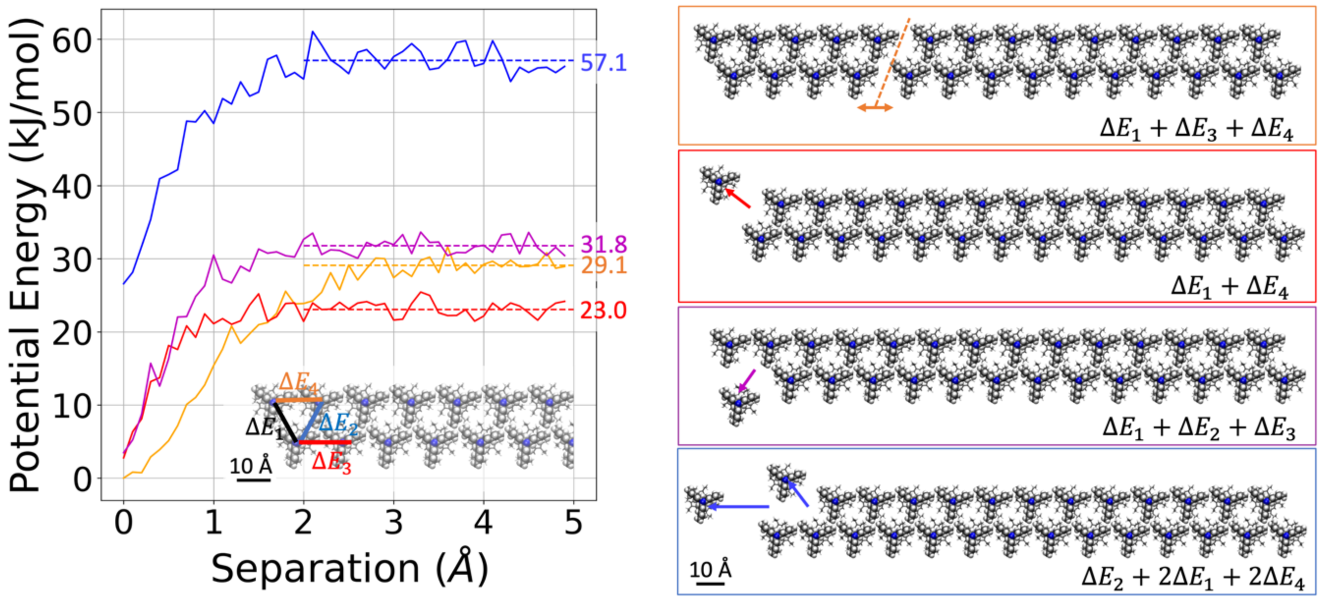

Supplementary Figure 12. Equivalent-spring constants from the realization of the daSSH model. From the dissociation energy plot, the interaction energies were calculated and equivalent springs constants determined. ΔE_1 = 14.73 kJ/mol, ΔE_2 = 10.99 kJ/mol, ΔE_3 = 6.04 kJ/mol, ΔE_4 = 8.33 kJ/mol. The atomistic top view in the inset starts with strong spring. The CHARMM potential energy was calculated every 0.1Å following the minimization algorithm L-BFGS.

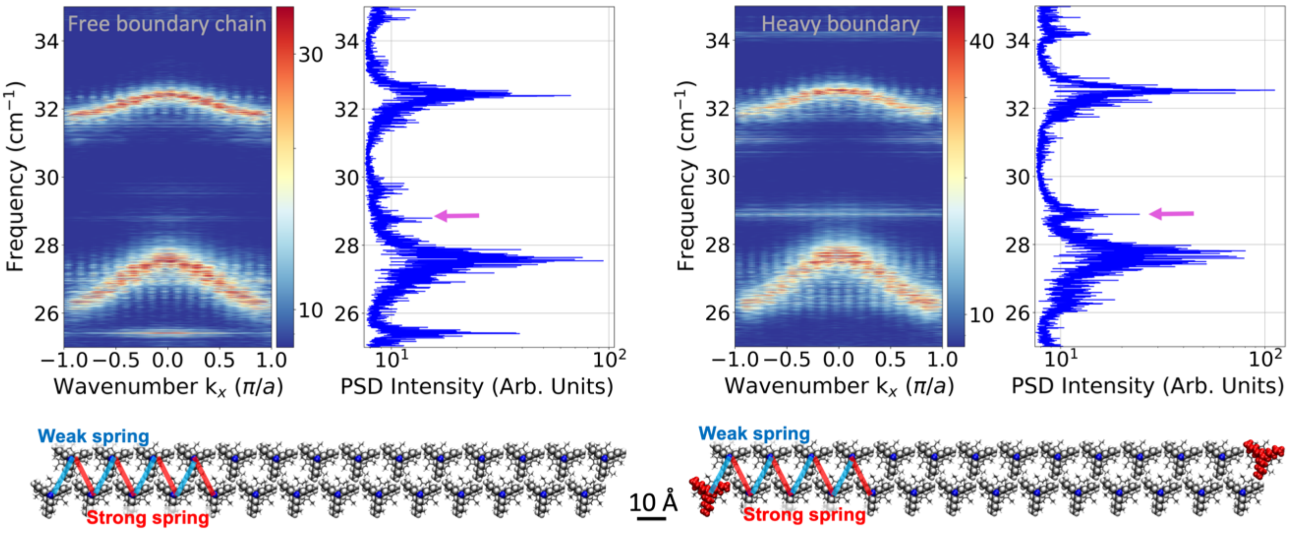

Supplementary Figure 13. Trivial case for the Figure 5 of the main text. In both cases, the molecular chain starts with a weak spring. No new boundary modes are detected upon creating a heavy boundary at the shown molecule in read. A boundary mode in both free boundary and heavy boundary is identified.

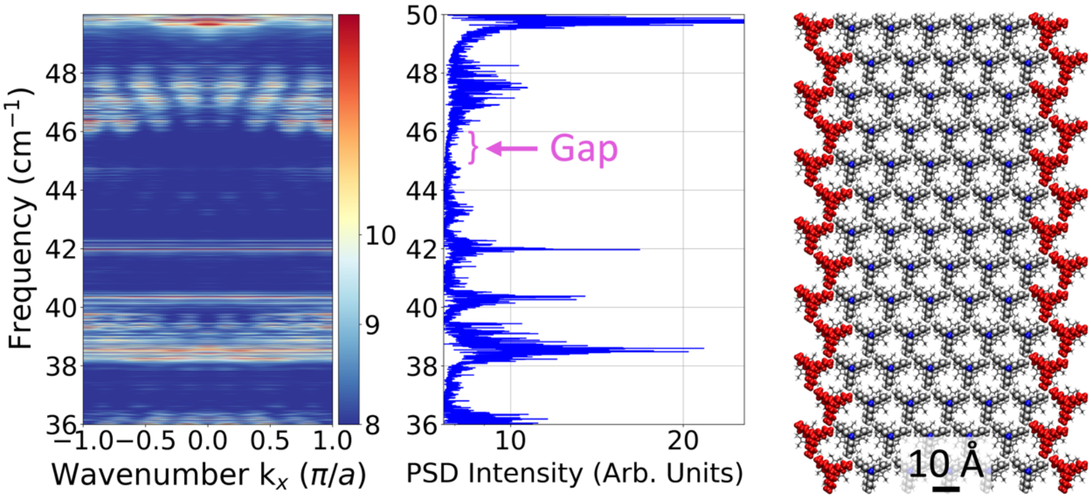

Supplementary Figure 14. Trivial case for the Figure 6 of the main text. Here the heavy boundary is next to a weak spring in alternating rows of the ribbon. We identified a gap in the region of interest.

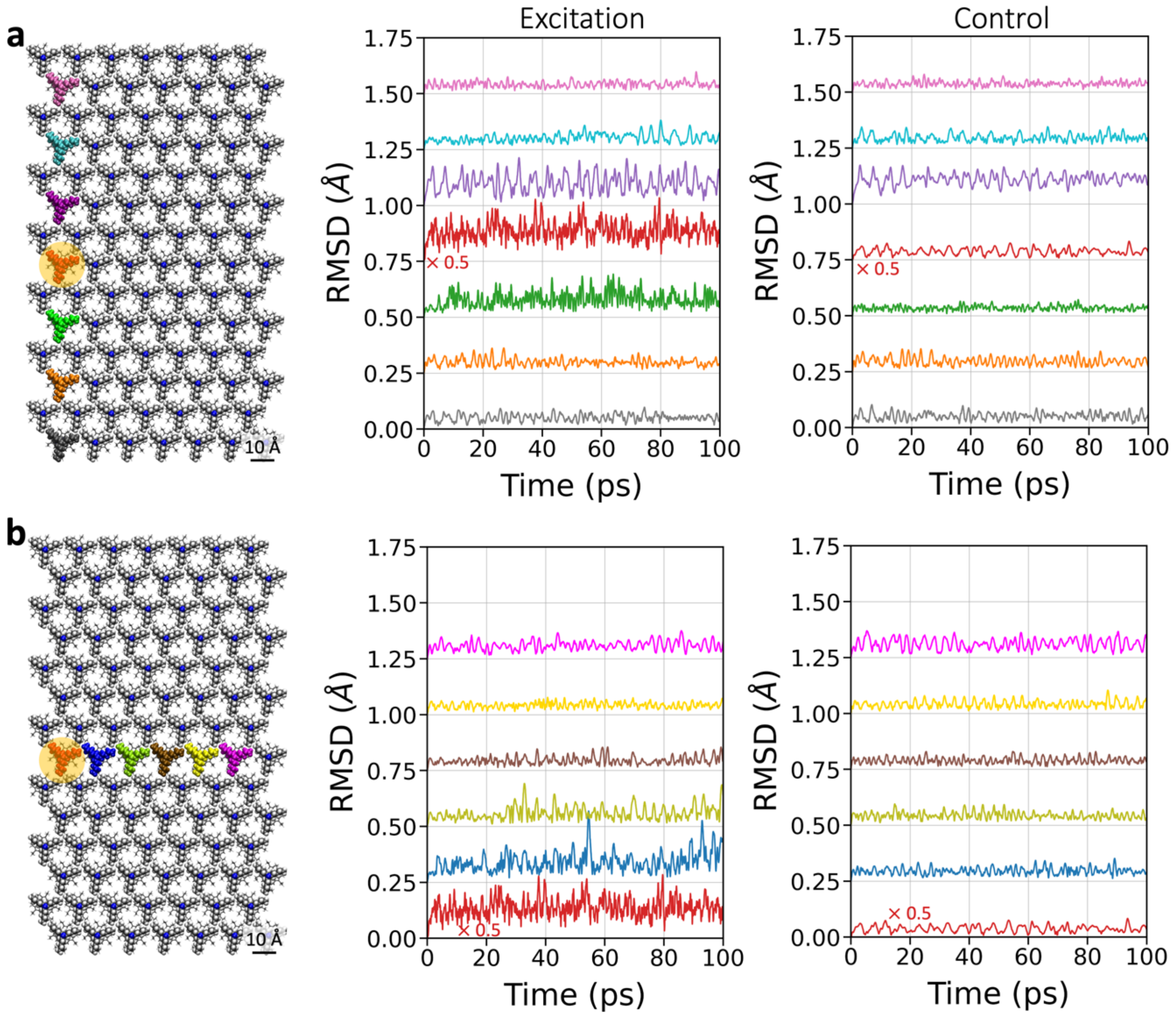

Supplementary Figure 15. Root mean square distance (RMSD) fluctuations on the coloured molecules as an effect of the excitation of the red molecule circled in yellow in the molecular diagrams. **a.** Along the boundary and **b.** along the bulk. Right panels show the control experiment using the same initial velocities as in the excitation. The MD simulation conditions are the same as in Figure 7 of the main text.

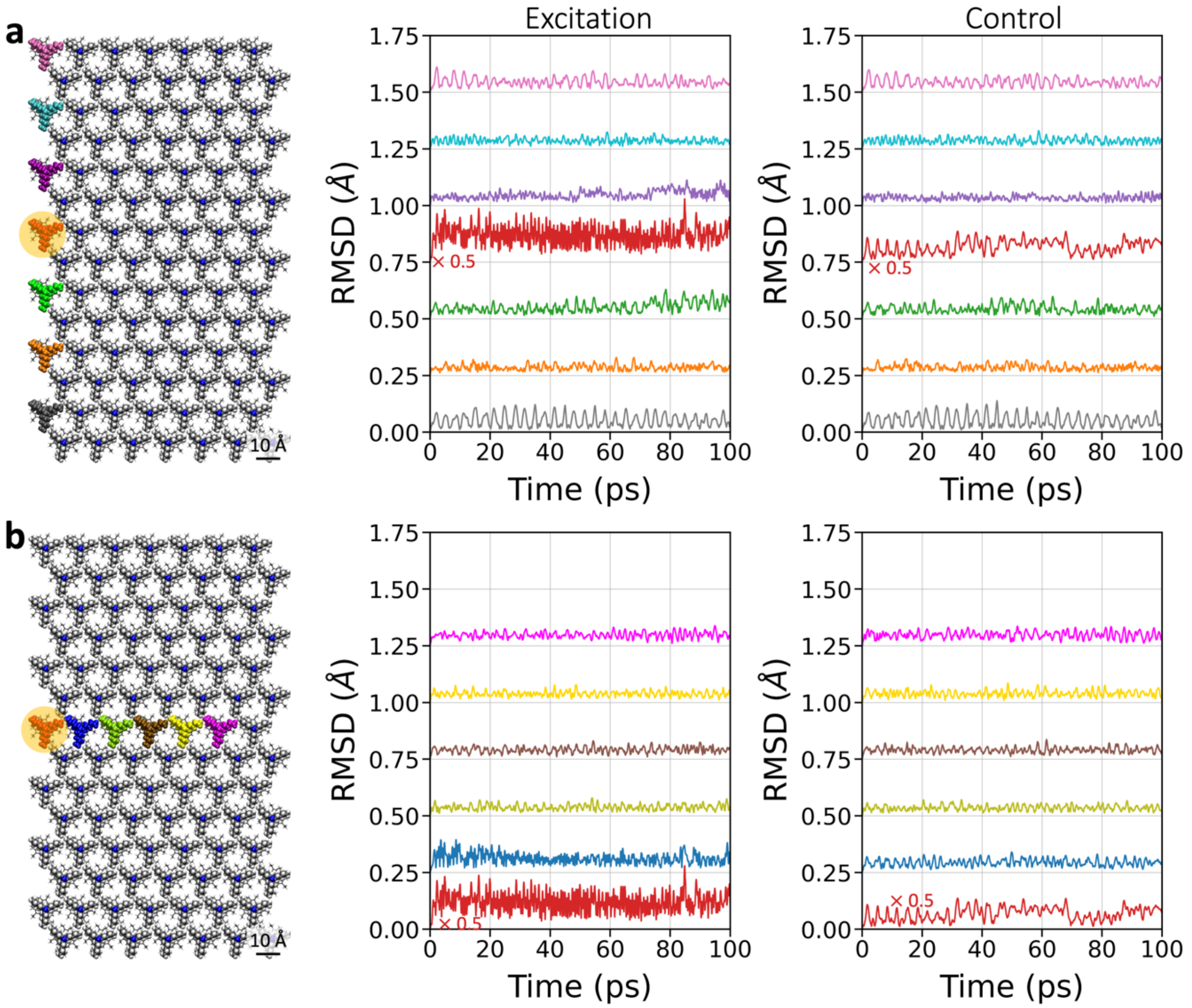

Supplementary Figure 16. RMSD fluctuations on the coloured molecules as an effect of the excitation of the red molecule circled in yellow in the molecular diagrams. **a.** Along the boundary and **b.** along the bulk. Right panels show the control experiment using the same initial velocities as in the excitation. The MD simulation conditions are the same as in Figure 8 of the main text.

References:

1. Lubensky, T. C., Kane, C. L., Mao, X., Souslov, A. & Sun, K. Phonons and elasticity in critically coordinated lattices. *Rep. Prog. Phys.* **78**, 073901 (2015).
2. Süsstrunk, R. & Huber, S. D. Classification of topological phonons in linear mechanical metamaterials. *Proc. Natl. Acad. Sci.* **113**, E4767–E4775 (2016).
3. Verlet, L. Computer 'Experiments' on Classical Fluids. I. Thermodynamical Properties of Lennard-Jones Molecules. *Phys. Rev.* **159**, 98–103 (1967).
4. Nosé, S. A molecular dynamics method for simulations in the canonical ensemble. *Mol. Phys.* **52**, 255–268 (1984).
5. Hoover, W. G. Canonical dynamics: Equilibrium phase-space distributions. *Phys. Rev. A* **31**, 1695–1697 (1985).
6. Wanko, M. *et al.* Polyyne electronic and vibrational properties under environmental interactions. *Phys. Rev. B* **94**, 195422 (2016).
