# Supplementary material for: Heavy-boundary mode patterning and dynamics of topological phonons in polymer chains and supramolecular lattices on surfaces
Source: Nat Commun. 2024 Dec 11;15:10674. doi: 10.1038/s41467-024-54511-8 (PMC11634973; doi:10.1038/s41467-024-54511-8)
Supplement: Supplementary file 3 — Description Of Additional Supplementary File [file 41467_2024_54511_MOESM3_ESM.pdf]

Supplementary Dataset

XYZ coordinates in XLSX sheets for the molecular structures represented in the Figures, corresponding to either initial geometrical optimisation for molecular dynamics (Figures 5a, 5b, 6a, 6b, 6c, 7a, 8d), geometrical optimisation for normal mode analysis (Figures 1g, 2b, 5c, 6e, 6i) or molecular dynamics snapshots (Figures 3b, 4d)
